# Association of quality antenatal care and completion of eight or more antenatal care visits with skilled delivery care utilization among pregnant women in Bangladesh: A nationwide population–based study

**DOI:** 10.1371/journal.pone.0322725

**Published:** 2025-04-29

**Authors:** Md. Obaidur Rahman, Md. Abdur Rauf, Yunefit Ulfa, Md. Nure Alam Siddiqi, Md. Rafiqul Islam, Kimiko Inaoka, Reiko Miyahara, Daisuke Yoneoka, Erika Ota

**Affiliations:** 1 Center for Evidence–Based Medicine and Clinical Research, Dhaka, Bangladesh; 2 Center for Surveillance, Immunization, and Epidemiologic Research, National Institute of Infectious Diseases, Tokyo, Japan; 3 Department of Global Health Nursing, St. Luke’s International University, Tokyo, Japan; 4 Department of Population Science and Human Resource Development, University of Rajshahi, Rajshahi, Bangladesh; 5 National Research and Innovation Agency, Jakarta, Indonesia; 6 Department of Population Science, Jatiya Kabi Kazi Nazrul Islam University, Mymensingh, Bangladesh; 7 Department of Global Health Nursing, International University of Health and Welfare, Chiba, Japan; Mizan-Tepi University, ETHIOPIA

## Abstract

**Introduction:**

Provision of quality antenatal care (QANC) services and delivery care by skilled health professionals can effectively reduce and manage complications throughout pregnancy and childbirth, leading to better maternal and neonatal health outcomes. The WHO recently updated its recommendation to at least eight antenatal care (ANC) visits. However, little is known about how QANC services and completion of eight or more ANC visits interact with skilled delivery care utilization.

**Methods:**

This study utilized data from Bangladesh Demographic and Health Survey 2017–18, including 4,457 pregnant women aged 15–49 years who had given birth three years preceding the survey. Descriptive statistics were employed to analyze the study population’s characteristics and the proportion of skilled birth attendance (SBA) and facility delivery (FD) in Bangladesh. Multilevel mixed–effects logistic regression analyses were used to determine the strength of association of QANC services and completion of eight or more ANC visits with skilled delivery care utilization.

**Results:**

Only one in five women received good QANC services, and one in eight completed eight or more ANC visits. The overall proportions of SBA and FD were 56.74% [95% CI: 55.27–58.20] and 53.85% [52.37–55.32] respectively. Women with eight or more ANC visits had significantly higher odds of utilizing SBA (OR: 2.11 [1.60–2.77]) and FD (OR: 2.19 [1.68–2.85]) compared to those with only 1–3 ANC visits. Likewise, good QANC services were associated with higher odds of SBA (OR: 1.72 [1.38–2.15]) and FD (OR: 1.56 [1.26–1.93]).

**Conclusion:**

This study highlights the significant positive association of QANC services and adherence to the WHO–recommended eight or more ANC visits with increased skilled delivery care utilization in Bangladesh. Strengthening policies and programs to enhance the quality and frequency of ANC services can promote skilled delivery care, ensuring safe motherhood and childbirth.

## Introduction

Globally, maternal and neonatal health remain critical public health concerns, particularly in low– and middle–income countries (LMICs), where maternal and neonatal mortality rates are still unacceptably high. Despite notable progress in recent years, maternal mortality continues to be a serious issue in many LMICs, including Bangladesh. In 2020, approximately 800 women died each day due to childbirth–related causes and pregnancy complications, with 95% of these deaths occurring in LMICs [1]. These deaths are largely preventable with consistent access to quality antenatal care (QANC) and skilled delivery services, both of which are essential for reducing and managing pregnancy complications and ensuring safer deliveries [2–4]. To address these challenges, the United Nations has set the Sustainable Development Goal (SDG) 3.1, which aims to reduce the global maternal mortality ratio to less than 70 per 100,000 live births by 2030, emphasizing the need for enhanced access to high–quality care during pregnancy, childbirth, and the postpartum period [5].

Antenatal care (ANC), the care women receive during pregnancy, is crucial for ensuring better health for both mother and their newborns. Such care includes regular health assessments, nutritional support, education on pregnancy complications, and delivery planning. These interventions offer detecting pregnancy–related risks early and ensuring timely medical care, thereby improving maternal and neonatal outcomes [4,6,7]. Recognizing the importance of frequent care during pregnancy, the World Health Organization (WHO) updated its recommendations in 2016 to advocate for at least eight ANC visits, up from the previous recommendation of four, to improve pregnancy outcomes [8]. The rationale behind increasing the number of ANC visits is to promote early detection of pregnancy complications, provide health education, ensure proper monitoring of maternal and fetal health, and create a plan for safe delivery. ANC visits also provide a critical opportunity to inform pregnant women about the importance of delivering in health facilities with the assistance of skilled birth attendants (SBAs). These professionals, such as doctors, nurses, and midwives, are trained to manage routine deliveries as well as obstetric emergencies, significantly reducing the risks of maternal and neonatal mortality. However, attending ANC visits without receiving quality care can lead to missed opportunities for early detection of complications and inadequate health promotion. A good quality ANC helps pregnant women in detecting and managing pregnancy complications effectively not only during the visits but also between the visits as women were counseled on danger signs of pregnancy during their visits. The component of QANC includes services such as weight and blood pressure measurements, ultrasound, blood and urine tests, iron supplementation, and education on pregnancy complications and management, although it may vary across countries [9,10]. These QANC services may increase the likelihood of a safe delivery by promoting early detection of pregnancy complications and treatment that can save the lives of the mother and newborn [11].

Like a high–burden LMIC, Bangladesh is still far from the SDG targets related to maternal and neonatal mortality, while an improvement, and faces several challenges in increasing both the coverage and quality of ANC and skilled delivery care services. The maternal mortality ratio in Bangladesh was reported as 123 deaths per 100,000 live births in 2020 [1]. Furthermore, only 47% of pregnant women received four or more ANC visits from any provider and even fewer (18%) women received QANC services in Bangladesh [12]. Although the WHO recommends a minimum of eight ANC visits for comprehensive maternal care [8], Bangladesh’s national guidelines still promote four or more ANC visits [13]. Furthermore, only 53% of deliveries were attended by skilled health professionals, and 49% births occur in health facilities [12]. These gaps in care highlight the need for interventions that not only increase the frequency of ANC visits but also improve the quality of care provided during pregnancy and childbirth.

Although several studies in LMICs, including Bangladesh, have explored factors associated with ANC utilization and delivery care, very few studies have examined the association of QANC services and the completion of eight or more ANC visits with skilled delivery care utilization [13–16]. Most studies have focused on the number of ANC visits, but little is known about how receiving high–quality ANC interacts with the updated WHO recommendations to improve the use of SBA and facility deliveries (FDs) [16–22]. Addressing this gap is essential for informing policies and interventions aimed at improving maternal health outcomes in high–burden LMICs like Bangladesh. Therefore, this study aimed to fill this gap by investigating the association between receiving QANC services and completing eight or more ANC visits with the utilization of SBA and FD among pregnant women in Bangladesh. The specific objectives of this study are to 1) estimate the proportion of women utilizing SBA and FD, stratified by their individual–, household–, and community–level factors; and 2) assess the strength of association of QANC services and completion of eight or more ANC visits with the utilization of SBA and FD in Bangladesh. This study will contribute to a deeper understanding of how the frequency and quality of ANC can influence skilled delivery care, providing valuable insights for policymakers to improve maternal and neonatal health outcomes in similar settings like Bangladesh.

## Materials and methods

### Data source and study population

This study utilized data from the 2017–18 Bangladesh Demographic and Health Survey (BDHS), a nationally representative cross–sectional survey of women aged 15–49 years. The BDHS employed a two–stage stratified cluster sampling design based on enumeration areas (EAs) identified in the 2011 Bangladeshi census. In the first stage, 675 EAs were selected with probability proportional to size, comprising 250 urban and 425 rural areas. Each EA, the primary sampling unit (PSU), contained an average of 120 households. In the second stage, a systematic sample of approximately 30 households per EA was selected. The detailed of the survey methodology can be found elsewhere [12].

A total of 20,250 households were selected, with an expectation to complete interviews with around 20,100 ever–married women aged 15–49 years. The survey was successfully conducted in 672 clusters after the exclusion of three clusters (one urban and two rural) degraded by floodwater, resulting in 20,160 households surveyed. Of these, 19,457 households were interviewed. Among 20,376 eligible ever–married women, 20,127 were interviewed (99% response rate), with non–response primarily due to the absence of women during home visits. For this study, we included pregnant women aged 15–49 years who had given birth within three years preceding the survey and had received at least one ANC visit during pregnancy. After excluding missing observations, the final analytical sample included 4,457 women aged 15–49 years (Supplementary Figure 1 in S1 File).

### Outcome variables

This study included two binary outcome variables: SBA at the time of delivery and FD. SBA refers to delivery care provided by a trained health professional (such as doctors, nurses, midwives, paramedics, community skilled birth attendants, or family welfare visitors), and this variable was coded as 1 if a skilled health professional assisted the delivery and 0 if the delivery occurred without any skilled assistance. The FD was coded as 1 if the delivery took place in a health facility (e.g., government or private hospitals, health centers, maternal and child welfare centers, NGO static clinics, or sub–district health centers) and 0 if the delivery was at home or in any non–institutional setting.

### Key explanatory variables

ANC visits: Women were asked about the frequency of ANC visits during their last pregnancy. Based on the WHO’s recommendation, we classified them into 4 categories: 1–3, 4, 5–7, and ≥ 8 visits.

QANC services: The QANC services were considered as an binary explanatory variables, coded as 1 (good QANC) if the pregnant women received all seven essential ANC components at least once and 2 (poor QANC) otherwise. These components include: measurement of weight, blood pressure, ultrasound, urine test, blood test, given or bought iron tablet, and counseling or information on signs of pregnancy complications during their ANC visits. Each component was assessed with a dichotomous response (1 = yes, 0 = no). The construction of this exposure variable was guided by the WHO ANC guidelines, ensuring alignment with recommended standards for comprehensive ANC.

Delay to the first ANC visit: The delay to the first ANC visits was coded as 0 (no) if the first ANC visits occurred within the first trimester (before 12 weeks of gestation), and coded as 1 (yes) if the first visit occurred during the second or third trimester.

ANC danger signs: Awareness of ANC danger signs was coded as 1 (yes) if a woman was informed or aware of at least one danger sign during her ANC visits, and coded as 0 (no) if she was not informed or aware of any danger signs.

### Covariates

Based on a rapid review of literature, we considered individual–, household–, and community–level factors and their respective categories. Individual–level factors include women’s age (15–24, 25–34, 35–49 years), education (primary or no formal education, secondary, higher), occupation (unemployed, employed), and body mass index (BMI: < 18.5, 18.5–24.9, 25.0–29.9, ≥ 30 kg/m^2^). Household–level factors comprise husband’s education (primary or no formal education, secondary, higher), husband’s occupation (agriculture, manual labor, business, services, others), children ever born (CEB: 1–2, ≥ 3), wealth index (poor/poorest, middle, rich/richest), decision on women’s healthcare (respondent alone, respondent and husband, someone else), distance to near health facilities (big problem, not a big problem), and intended pregnancy (yes, no). Community–level factors include religion (non–Muslim, Muslim), place of residence (urban, rural), and division (Barisal, Chittagong, Dhaka, Khulna, Mymensingh, Rajshahi, Rangpur, Sylhet).

### Statistical analysis

Descriptive statistics were employed to analyze the study population’s characteristics and the proportion of SBA and FD, stratified by the individual–, household–, and community–level factors. A chi–squared test was used to ascertain the association of outcome variables (SBA and FD) with individual–, household–, and community–level exposure variables. Multilevel analysis, specifically a two–level mixed–effects logistic regression model, was performed to determine the strength of association of QANC services and completing eight or more ANC visits with the outcome variables, accounting for the hierarchical structure of the 2017–18 BDHS data where individuals were nested within communities. We fitted five models: the null model (Model 0), Model 1, Model 2, Model 3, and Model 4. The null model included only the outcome variable to establish baseline variance. Model 1 was fitted for key explanatory variables and this model was subsequently adjusted by adding individual–level factors (Model 2), household–level factors (Model 3), and community–level factors (Model 4) sequentially. The results of fixed–effect estimates were reported as adjusted odds ratios (OR) with their 95% confidence intervals (CIs). The random effects in the estimated intercepts within communities were evaluated using the intra–class correlation coefficient (ICC) to compare the variance across models. Multicollinearity was also assessed using variance inflation factors (VIF), and no multicollinearity issues were identified (all VIF < 2, the average VIF of 1.31). Model fitting performance was evaluated using Akaike Information Criterion (AIC) and Bayesian Information Criterion (BIC), with the lower values indicating the better model. Statistical significance was defined as p < 0.05, and all analyses were performed using STATA version 18–MP (StataCorp, Texas, USA) [23].

### Ethical approval

This study did not require ethical approval as it involved the analysis of secondary data of the 2017–18 BDHS, which was obtained from the DHS program following the submission of the study protocol. The DHS data collection procedures received approval from the ICF Institutional Review Board, and obtained written informed consent from all participants before their enrolment. The custodian of the DHS Program provided the data in deidentified form, ensuring the anonymity and confidentiality of all participants.

## Results

### Background characteristics of the study population

**Fig 1:**
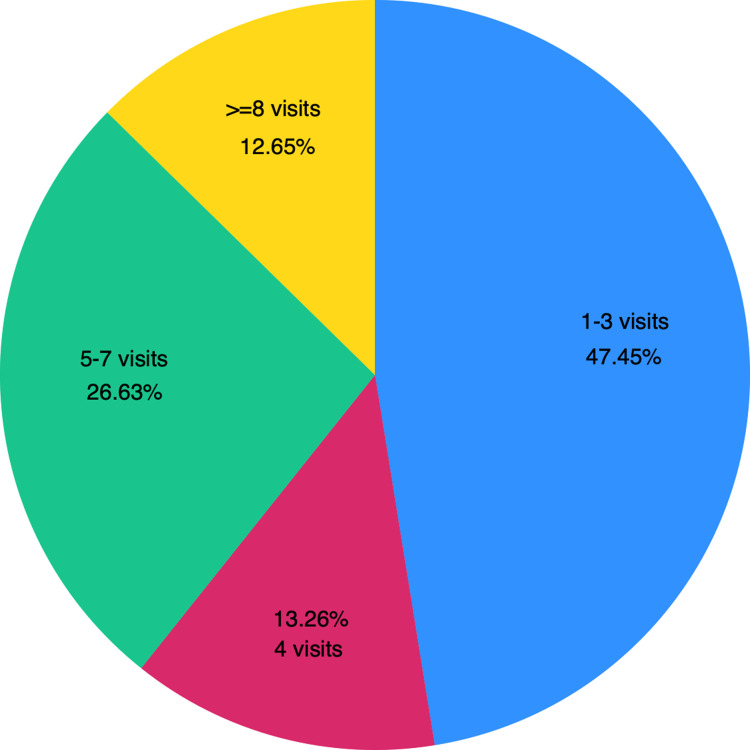
Proportion of women with completion of antenatal care visits, BDHS 2017–18.

**Fig 2:**
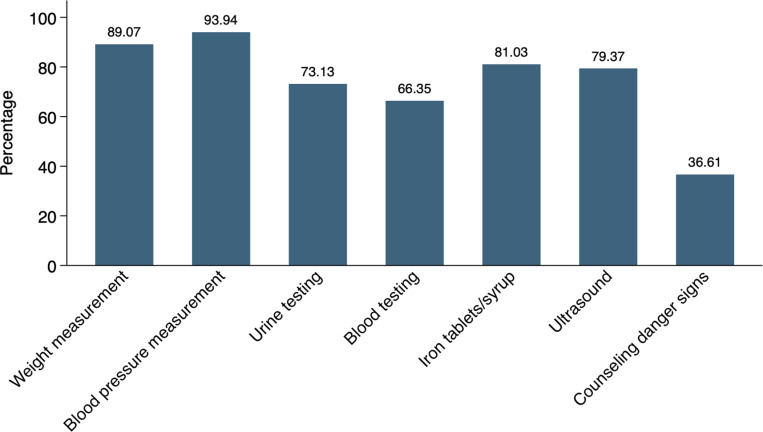
Coverage of antenatal care service components, BDHS 2017–18.

A total of 4,457 pregnant women aged 15–49 years who had given birth within three years preceding the survey were included in the final analysis (Table 1, Supplementary Figure 1 in S1 File). Among these women, 47.45% completed 1–3 ANC visits, 13.26% completed exactly 4 ANC visits, 26.63% completed 5–7 ANC visits, and 12.65% completed eight or more ANC visits (Fig 1). Only 21.56% women received good QANC services whereas the majority (78.44%) received poor QANC services. Regarding the provision of the seven essential components of ANC, the measurement of blood pressure was the most commonly provided service, received by 93.94% pregnant women, followed by weight measurement (89.07%), ultrasound (79.37%), urine testing (73.13%), and blood testing (66.35%). Furthermore, 81.03% women received iron tablets/syrups and 36.61% were informed about danger signs during their ANC visits (Fig 2).

### Proportion of SBA and FD utilization by individual–, household–, and community–level characteristics

Table 1 presents the proportion of SBA and FD utilization among pregnant women, stratified by individual–, household–, and community–level characteristics. Overall, the proportions of SBA and FD were estimated at 56.74% [95% CI: 55.27–58.20] and 53.85% [52.37–55.32] respectively. Women aged 15–24 years had slightly higher proportions of SBA (58.18%) and FD (54.92%) compared to other age groups. Non–Muslim women reported higher proportions of SBA (65.65%) and FD (64.89%) than Muslim women, and urban residents had significantly higher rates (SBA 69.27%, FD 65.90%) compared to their rural counterparts. Women with a rich wealth index had the highest rates of SBA (75.13%) and FD (71.79%), and those with higher education levels reported the highest utilization rates. The proportions of SBA and FD were notably higher among those with a BMI ≥ 30 kg/m^2^ (SBA 80.38%, FD 77.03%).

**Table 1:** Proportion of skilled birth attendance (SBA) and facility delivery (FD), stratified by socio–demographic and health related characteristics, BDHS 2017–18.

Variables	N	SBA		FD	
		Proportion (95% CI)	P–value	Proportion (95% CI)	P–value
**Overall**	4,457	56.74 (55.27–58.20)		53.85 (52.37–55.32)	
**Women age** **(in years)**			0.115		0.29
15–24	2,358	58.18 (56.16–60.19)		54.92 (52.88–56.94)	
25–34	1,838	55.01(52.70–57.30)		52.77 (50.46–55.08)	
35–49	261	55.94 (49.69–62.06)		51.72 (45.48–57.93)	
**Religion**			0.000		0.000
Non–Muslim	393	65.65 (60.72–70.34)		64.89 (59.94–69.60)	
Muslim	4,064	55.88 (54.34–57.42)		52.78 (51.23–54.33)	
**Residence**			0.000		0.000
Urban	1,572	69.27 (66.93–71.55)		65.90 (63.50–68.25)	
Rural	2,885	49.91 (48.07–51.75)		47.28 (45.44–49.12)	
**Division**			0.000		0.000
Barisal	447	55.70 (50.96–60.37)		46.53 (41.83–51.28)	
Chittagong	728	55.36 (51.66–59.01)		51.51 (47.81–55.20)	
Dhaka	656	64.94 (61.15–68.59)		62.65 (58.82–66.37)	
Khulna	486	67.28 (62.91–71.44)		64.40 (59.97–68.66)	
Mymensingh	535	47.85 (43.55–52.18)		45.42 (41.14–49.75)	
Rajshahi	492	59.76 (55.27–64.12)		58.13 (53.63–62.53)	
Rangpur	525	54.10 (49.72–58.42)		53.71 (49.34–58.04)	
Sylhet	588	49.32 (45.21–53.44)		47.96 (43.86–52.08)	
**Wealth index**			0.000		0.000
Poor	1,747	37.44 (35.16–39.75)		35.20 (32.96–37.49)	
Middle	824	55.58 (52.11–59.01)		52.31 (48.83–55.76)	
Rich	1,886	75.13 (73.12–77.07)		71.79 (69.70–73.81)	
**Women education**			0.000		0.000
Primary or no education	1,396	37.25 (34.71–39.85)		35.24 (32.73–37.81)	
Secondary	2,195	58.59 (56.49–60.66)		55.40 (53.29–57.49)	
Higher	866	83.49 (80.84–85.90)		79.91 (77.08–82.53)	
**Husband education**			0.000		0.000
Primary or no education	2,005	42.19 (40.02–44.39)		39.75 (37.60–41.93)	
Secondary	1,529	60.43 (57.93–62.89)		57.29 (54.77–59.79)	
Higher	923	82.23 (79.61–84.65)		78.76 (75.98–81.36)	
**Women occupation**			0.000		0.000
Unemployed	2,721	62.11 (60.26–63.94)		59.46 (57.59–61.32)	
Employed	1,736	48.33 (45.95–50.71)		45.05 (42.69–47.42)	
**Husband occupation**			0.000		0.000
Agriculture	830	41.81 (38.43–45.25)		39.28 (35.94–42.69)	
Manual labor	1,743	53.47 (51.10–55.83)		50.72 (48.34–53.09)	
Business	862	62.06 (58.73–65.32)		58.82 (55.45–62.13)	
Services	592	59.12 (55.04–63.11)		56.42 (52.32–60.46)	
Others^*^	430	84.88 (81.14–88.14)		81.16 (77.14–84.75)	
**Body mass index** (kg/m^2^)			0.000		0.000
<18.5	672	45.98 (42.16–49.84)		44.35 (40.55–48.19)	
18.5–24.9	2,729	52.91 (51.02–54.80)		49.80 (47.91–51.69)	
25.0–29.9	847	71.78 (68.62–74.79)		68.71 (65.47–71.82)	
≥30	209	80.38 (74.34–85.54)		77.03 (70.73–82.55)	
**Intended pregnancy**			0.000		0.000
Yes	3,530	58.73 (57.08–60.36)		55.92 (54.26–57.57)	
No	927	49.19 (45.93–52.46)		45.95 (42.71–49.23)	
**Children ever born**			0.000		0.000
1–2	3,233	61.74 (60.4–63.42)		58.56 (57.14–60.56)	
≥3	1,224	43.55 (40.75–46.38)		40.60 (37.84–43.42)	
**Decision on women’s healthcare**			0.036		0.095
Respondent alone	337	62.61 (57.20–67.79)		57.57 (52.09–62.90)	
Respondent and husband	2,910	56.87 (55.05–58.68)		54.36 (52.53–56.19)	
Someone else	1,210	54.79 (51.94–57.62)		51.57 (48.71–54.42)	
**Distance to health facility**			0.000		0.000
Big problem	1,769	49.86 (47.50–52.22)		46.98 (44.63–49.33)	
Not a big problem	2,688	61.27 (59.40–63.12)		58.37 (56.48–60.24)	
**Number of ANC visits**			0.000		0.000
1–3	2,115	42.65 (40.53–44.79)		39.91 (37.81–42.03)	
4	591	61.59 (57.53–65.53)		58.38 (54.28–62.38)	
5–7	1,187	69.84 (67.14–72.44)		66.55 (63.7–69.24)	
≥8	564	76.95 (73.25–80.37)		74.65 (70.84–78.19)	
**Delay to first ANC visit**			0.000		0.000
No	1,854	68.72 (66.55–70.82)		65.64 (63.43–67.80)	
Yes	2,603	48.21(46.28–5.15)		45.45 (43.52–47.38)	
**Danger signs during pregnancy**			0.000		0.000
No	2,642	51.14 (49.21–53.06)		48.18 (46.26–50.11)	
Yes	1,815	64.90 (62.66–67.10)		62.09 (59.82–64.33)	
**QANC services**			0.000		0.000
Poor	3,496	51.09 (49.42–52.76)		48.37 (46.70–50.04)	
Good	961	77.32 (74.53–79.93)		73.78 (70.87–76.53)	

Note: SBA = Skilled Birth Attendance, FD = Facility Delivery, ANC = Antenatal Care, CI = Confidence Interval, QANC = Quality ANC.

P–value is estimated by Chi–square test.

*Others include professionals/technical/managerial and clerical personnels.

The proportions of SBA and FD increased with the number of ANC visits, peaking at 76.95% and 74.65% respectively, among women with eight or more ANC visits. The rates were significantly higher among women receiving good QANC services (SBA 77.32%, FD 73.78%, respectively) compared to those receiving poor QANC services (SBA 51.09%, FD 48.37%). Women who had no delay to their first ANC visit had higher SBA (68.72%) and FD (65.64%) rates compared to those who delayed (SBA 48.21%, FD 45.45%). Furthermore, women who experienced danger signs during pregnancy (SBA: 64.90%, FD: 62.09%), had 1–2 previous childbirths (SBA: 61.74%, FD: 58.56%), made healthcare decisions alone (SBA: 62.61%, FD: 57.57%), and reported no issues with distance to health facilities (SBA: 61.27%, FD: 58.37%) exhibited higher utilization rates. Significant differences were also found across various regions (divisions) and other factors.

### Association of SBA with the utilization of QANC services and completion of eight or more ANC visits

The association of SBA with the utilization of QANC services and completion of eight or more ANC visits was examined using multilevel mixed–effects logistic regression analysis, with results reported as adjusted OR and 95% CIs (Table 2). The goodness of fitting of our fully adjusted model (Model 4), with an AIC of 4952.897 and a BIC of 5196.182, suggest a good fitting performance. The model revealed that women with four ANC visits had 54% higher odds of having SBA compared to those with 1–3 visits (OR: 1.54 [1.23–1.93]). The odds of SBA increased further for women with 5–7 visits (OR: 1.94 [1.60–2.35]) and eight or more visits (OR: 2.11 [1.60–2.77]), indicating a dose–response relationship. Good QANC services were associated with significantly higher odds of SBA compared to poor QANC services (OR: 1.72 [1.38–2.15]). Conversely, a delay to the first ANC visit was associated with a reduction in the likelihood of SBA (OR: 0.83 [0.70–0.98]). However, no significant association was observed between ANC danger signs and SBA (OR: 1.06 [0.89–1.26]). The cluster–level variance was 0.40 [95% CI: 0.27–0.59], with an ICC of 10.82%, indicating that about 11% of the variance in SBA was due to differences between clusters. These findings highlight the importance of frequent, timely, and good–quality ANC for improving SBA outcome.

**Table 2:** Adjusted odds ratios and 95% confidence intervals (CIs) for skilled birth attendance (SBA) from multilevel mixed–effects logistic regression model.

Outcome: SBA	Model 0	Model 1	Model 2	Model 3	Model 4
**ANC visits**					
1–3 [ref]					
4		1.84 [1.48, 2.28]**	1.72 [1.38, 2.15]**	1.59 [1.27, 1.99]**	1.54 [1.23, 1.93]**
5–7		2.33 [1.93, 2.80]**	2.14 [1.78, 2.59]**	2.01 [1.66, 2.44]**	1.94 [1.60, 2.35]**
≥8		2.88 [2.21, 3.76]**	2.51 [1.91, 3.29]**	2.25 [1.71, 2.96]**	2.11 [1.60, 2.77]**
**QANC services**					
Poor [ref]					
Good		2.32 [1.87, 2.88]**	1.93 [1.55, 2.41]**	1.74 [1.39, 2.17]**	1.72 [1.38, 2.15]**
**Delay to the first ANC visit**					
No [ref]					
Yes		0.67 [0.57, 0.78]**	0.79 [0.67, 0.93]**	0.85 [0.72, 1.00]*	0.83 [0.70, 0.98]*
**ANC danger signs**					
No [ref]					
Yes		1.09 [0.92, 1.28]	1.06 [0.90, 1.26]	1.06 [0.89, 1.26]	1.06 [0.89, 1.26]
Cluster–level variance	1.14 [0.91, 1.43]	0.82 [0.63, 1.06]	0.53 [0.38, 0.74]	0.45 [0.31, 0.65]	0.40 [0.27, 0.59]
Intra–class correlation	25.76%	19.93%	13.89%	12.09%	10.82%
AIC	5779.117	5423.497	5101.676	4971.089	4952.897
BIC	5791.922	5474.715	5204.112	5156.753	5196.182

** p < .01, * p < .05.

Model 0: Null model.

Model 1: Adjusted for ANC visits, QANC services, delay to the first ANC visit, and ANC danger signs.

Model 2: Further adjusted for women’s age, education, occupation, and BMI.

Model 3: Further adjusted for husband’s education, husband’s occupation, CEB, wealth index, decision on women’s healthcare, distance to near health facilities, and intended pregnancy.

Model 4: Further adjusted for religion, place of residence, and division.

Note: AIC = Akaike Information Criterion, ANC = Antenatal Care, BIC = Bayesian Information Criterion, CI = Confidence Interval, QANC = Quality ANC, SBA = Skilled Birth Attendance.

### Association of FD utilization with QANC services and completion of eight or more ANC visits

Table 3 presents the results from our fully adjusted model for FD outcome (Model 4). The goodness of fitting of Model 4, with an AIC of 5080.383 and a BIC of 5323.668, suggest a good fitting performance. The model identified that women with four ANC visits had a significantly higher likelihood of FD compared to those with 1–3 visits (OR: 1.51 [1.21–1.88]). The odds of FD further increased for those with 5–7 visits (OR: 1.90 [1.57–2.29]), with the highest odds observed among women with eight or more visits (OR: 2.19 [1.68–2.85]). Good QANC services were significantly associated with higher odds of FD (OR: 1.56 [1.26–1.93]). Unlike SBA, a delay in the first ANC visit was not significantly associated with FD (OR: 0.87 [0.74–1.02]). Furthermore, the presence of ANC danger signs was not significantly associated with FD (OR: 1.09 [0.93–1.29]). Random effects analysis showed moderate variability at the cluster level, with a cluster–level variance of 0.34 [95% CI: 0.22–0.52] and an ICC of 9.45%, indicating that about 9% of the variance in FD was due to cluster differences. These findings emphasize the crucial role of frequent, good–quality ANC in promoting FD utilization among pregnant women.

**Table 3:** Adjusted odds ratios and 95% confidence intervals (CIs) for facility delivery (FD) from multilevel mixed–effects logistic regression model.

Outcome: FD	Model 0	Model 1	Model 2	Model 3	Model 4
**ANC visits**					
1–3 [ref]					
4		1.82 [1.47, 2.25]**	1.71 [1.38, 2.13]**	1.58 [1.27, 1.97]**	1.51 [1.21, 1.88]**
5–7		2.28 [1.90, 2.74]**	2.13 [1.77, 2.56]**	1.99 [1.65, 2.40]**	1.90 [1.57, 2.29]**
≥8		2.96 [2.29, 3.82]**	2.60 [2.00, 3.38]**	2.35 [1.80, 3.06]**	2.19 [1.68, 2.85]**
**Quality of ANC**					
Poor [ref]					
Good		2.09 [1.70, 2.57]**	1.75 [1.41, 2.16]**	1.57 [1.27, 1.95]**	1.56 [1.26, 1.93]**
**Delay to the first ANC visit**					
No [ref]					
Yes		0.69 [0.59, 0.81]**	0.82 [0.70, 0.96]*	0.88 [0.75, 1.03]	0.87 [0.74, 1.02]
**ANC danger signs**					
No [ref]					
Yes		1.12 [0.95, 1.32]	1.10 [0.93, 1.30]	1.09 [0.92, 1.29]	1.09 [0.93, 1.29]
Cluster–level variance	1.02 [0.81, 1.29]	0.73 [0.55, 0.95]	0.49 [0.35, 0.68]	0.41 [0.28, 0.60]	0.34 [0.22, 0.52]
Intra–class correlation	23.71%	18.06%	12.94%	11.11%	9.45%
AIC	5868.226	5526.062	5225.352	5109.072	5080.383
BIC	5881.030	5577.280	5327.788	5294.737	5323.668

** p < .01, * p < .05.

Model 0: Null model.

Model 1: Adjusted for ANC visits, QANC services, delay to the first ANC visit, and ANC danger signs.

Model 2: Further adjusted for women’s age, education, occupation, and BMI.

Model 3: Further adjusted for husband’s education, husband’s occupation, CEB, wealth index, decision on women’s healthcare, distance to near health facilities, and intended pregnancy.

Model 4: Further adjusted for religion, place of residence, and division.

Note: AIC = Akaike Information Criterion, ANC = Antenatal Care, BIC = Bayesian Information Criterion, CI = Confidence Interval, FD = Facility Delivery, QANC = Quality ANC.

## Discussion

To the best of our knowledge, this is the first study in its nature providing comprehensive insights into the strength of association of QANC services and the completion of eight or more ANC visits with skilled delivery care utilization among pregnant women in Bangladesh. Our findings indicate that women who completed eight or more ANC visits had significantly higher odds of utilizing SBA and FD compared to those with fewer visits, demonstrating a clear dose–response relationship. This aligns with the WHO’s recommendation of a minimum of eight ANC visits to improve maternal and neonatal health outcomes [8]. The findings suggest that increased frequency of ANC visits enhances maternal engagement with healthcare services, fosters trust in the healthcare system, and encourages the use of skilled delivery care. Similar associations between higher ANC visits and skilled delivery care utilization have been reported in studies from other LMICs, including Ghana, Tanzania, Ethiopia, and Nigeria [15,24–32], reinforcing the broader applicability of these findings. Furthermore, a study conducted in Bangladesh, utilizing data from the 2014 BDHS, demonstrated a positive association between the frequency of ANC visits and the likelihood of accessing SBAs and delivering in health facilities [15]. Another study analyzed data across 19 LMICs found that women who attended eight or more ANC visits had a 14% higher chance of delivering at health facilities compared to those who attended less than eight ANC visits [30]. These findings are consistent with our findings.

The positive association of QANC services with skilled delivery care utilization further highlights the importance of the content and quality of ANC visits. Women who received good QANC services, encompassing essential components like blood pressure measurement, ultrasound, and danger sign counseling, were significantly more likely to utilize SBA and FD. These findings suggest that the quality of care, not just the quantity, plays a pivotal role in influencing maternal health behaviors. Good QANC services provide opportunities for early detection and management of pregnancy–related complications, education on birth preparedness, and timely referrals, which are crucial for ensuring safe delivery outcomes. The findings of our study echo previous research that links high QANC services to improved maternal health–seeking behaviors and better childbirth experiences [33–36]. For instance, a study conducted in Bangladesh emphasized the importance of QANC services in promoting maternal health and enhancing birth outcomes [14]. Another study across 28 African countries reported that higher QANC services predict retention in SBA at the time of delivery [36], which supports our study findings.

Our study noticed that while more than half of the women received skilled delivery care, only a small proportion of women in Bangladesh accessed good QANC services (21.56%) and completed the recommended eight or more ANC visits (12.65%). Furthermore, a clear dose–response interaction between ANC visits and QANC services was demonstrated in our further analysis (Supplementary Table 1 in S1 File). Urban women with higher education and wealth were more likely to receive frequent ANC visits, QANC services, SBA, and deliver at health facilities compared to rural women, aligning with studies in Bangladesh [37] and African countries such as Sub–Saharan Africa, Ghana, and Guinea [38–40]. The concentration of public and private healthcare facilities in urban areas provides easier access urban women, highlighting the need to bridge the urban–rural gap by establishing comprehensive referral hospitals, deploying healthcare workers and midwives to rural areas, and implementing community–based health programs [41]. Financial constraints, limited access to health facilities, and sociocultural factors often hinder rural women from completing the recommended ANC visits and receiving comprehensive care throughout pregnancy. Therefore, strategies to enhance ANC utilization should include community–based education, improved health facility accessibility, male involvement, and policies that address financial barriers to healthcare.

The association between delayed initiation of ANC visits and reduced odds of SBA further emphasizes the importance of early engagement with ANC services [42]. Women who initiated ANC in the first trimester were more likely to utilize skilled delivery care, reflecting the benefits of timely access to health information and services. Early ANC visits provide critical opportunities for health education, identification of high–risk pregnancies, and planning for skilled delivery care. Factors contributing to delayed initiation of ANC visits include lack of awareness, financial constraints, and cultural beliefs [43,44]. Addressing these barriers through targeted interventions, such as raising awareness about early ANC initiation and modifying socio–cultural norms, is essential. These efforts could contribute to improved maternal and neonatal health outcomes by encouraging early ANC engagement and increasing the likelihood of SBA. However, our analysis in the context of Bangladesh found no association between the delayed ANC initiation and FD utilization, suggesting that other factors like financial burdens may influence FD use in this setting. This finding underscores the importance of context–specific strategies to promote facility–based delivery, considering the unique barriers and facilitators that vary across different regions.

Women identified with danger signs during ANC visits may require specialized care and interventions to mitigate risks during childbirth. However, our study found no significant association between the presence of ANC danger signs and the utilization of SBA or FD. This finding contrasts with other studies, suggesting that danger signs identified during ANC visits can motivate women to seek skilled delivery care [41,45]. To address these challenges, it is crucial to strengthen community–based education and awareness programs, enhance the capacity of frontline healthcare providers to recognize and manage pregnancy complications, and improve the availability of emergency obstetric care services.

The strengths of this study include the use of nationally representative data and a large sample size, which enhance the reliability and precision of the results. Additionally, the use of a multilevel mixed–effects logistic regression model accounts for the clustered nature of the data, allowing for the examination of variations within and between clusters, and providing robust and generalizable findings applicable to similar settings like Bangladesh. However, our findings should be interpreted with caution. The cross–sectional nature of the BDHS data limits causal interpretations of the observed associations. While the dataset remains relevant due to its nationally representative nature, we acknowledge that its age is a limitation. The survey was conducted before the COVID-19 pandemic, and during which the healthcare system and services were significantly disrupted. Therefore, more recent investigations, particularly post–pandemic, are needed to confirm our findings. The reliance on self–reported data for ANC visits, QANC components, and delivery care utilization may introduce recall bias, although the large sample size and high response rate mitigate some of these limitations. Additionally, our definition of good QANC services is limited, as it primarily captures whether centain components of care (e.g., blood pressure measurement) were performed but does not assess whether appropriate follow–up actions were taken in response to abnormal findings. This distinction is crucial for assessing the true quality of care, and future research should adopt a more comprehensive approach to evaluating good QANC services. Our study observed that around half of the women did not receive skilled delivery care services; however, we could not explore their underlying causes due to limitation of the BDHS dataset. Furthermore, our study did not explore potential barriers to accessing services, such as financial constraints, cultural factors, or geographical accessibility, which could provide further insights for targeted interventions.

## Conclusion

This study demonstrates a significant positive association of QANC services and completion of eight or more ANC visits with the utilization of SBA and FD among pregnant women in Bangladesh. The findings underscore critical policy implications, emphasizing the need to prioritize both the quality and frequency of ANC visits in efforts to improve maternal and neonatal health outcomes. Policymakers should focus on enhancing ANC service delivery to ensure all pregnant women have access to high–quality, comprehensive care throughout pregnancy. Promoting frequent and timely ANC visits, along with good–QANC services, can significantly increase skilled delivery care utilization, leading to safer childbirth and better maternal and neonatal health outcomes in Bangladesh and similar settings. Future research should explore barriers to ANC use and skilled delivery care utilization, and evaluate targeted interventions to address these challenges, supporting the SDG of reducing maternal and neonatal mortality and promoting safe motherhood and childbirth.

## Supporting information

S1 File Supplementary appendix. (DOCX)

S2 File STROBE statement-checklist for observational studies. (DOCX)
