# Supplementary material for: Association of quality antenatal care and completion of eight or more antenatal care visits with skilled delivery care utilization among pregnant women in Bangladesh: A nationwide population–based study
Source: PLoS One. 2025 Apr 29;20(4):e0322725. doi: 10.1371/journal.pone.0322725 (PMC12040161; doi:10.1371/journal.pone.0322725)
Supplement: S1 File — (DOCX) [file pone.0322725.s001.docx]

**Association of quality antenatal care and completion of eight or more antenatal care visits with skilled delivery care utilization among pregnant women in Bangladesh: A nationwide population–based study**

Md. Obaidur Rahman, Md. Abdur Rauf, Yunefit Ulfa, Md. Nure Alam Siddiqi, Md. Rafiqul Islam, Kimiko Inaoka, Reiko Miyahara, Daisuke Yoneoka, Erika Ota

**Supplementary appendix**

**Supplementary Table 1. Association between quality antenatal care (QANC) services and antenatal care (ANC) visits by multilevel mixed–effects logistic regression model**

| **Outcome: QANC services** | **Model 0** | | **Model 1** | |
| --- | --- | --- | --- | --- |
|  | **OR (95% CI)** |  | **OR (95% CI)** |  |
| **ANC visits** |  |  |  |  |
| 1–3 [ref] |  |  |  |  |
| 4 |  |  | 2·21 | ** |
|  |  |  | [1·71, 2·86] |  |
| 5–7 |  |  | 3·73 | ** |
|  |  |  | [3·03, 4·59] |  |
| >=8 |  |  | 5·26 | ** |
|  |  |  | [4·07, 6·80] |  |
| **Delay to the first ANC visit** |  |  |  |  |
| No [ref] |  |  |  |  |
| Yes |  |  | 0·73 | ** |
|  |  |  | [0·61, 0·87] |  |
| Cluster–level variance | 0·50 |  | 0·39 |  |
|  | [0·35, 0·72] |  | [0·25, 0·59] |  |
| Intra–class correlation | 13·26% |  | 10·51% |  |
| AIC | 4584·805 |  | 4257·764 |  |
| BIC | 4597·61 |  | 4296·177 |  |
| ** p<·01, * p<·05  Model 0: Null model  Model 1: Adjusted for ANC visits and delay to the first ANC visit | | | | |
| Note: AIC= Akaike Information Criterion, ANC= Antenatal Care, BIC=Bayesian Information Criterion, CI=Confidence Interval, OR=Odds Ratio, QANC= Quality ANC | | | | |

20,160 households selected from 672 enumeration areas in BDHS 2017-18

19,457 households interviewed (99.4% response rate)

20,376 ever-married women eligible for interview

20,127 ever-married women were interviewed (99.4% response rate)

5,007 women gave birth within three years preceding the survey

4,457 women included in this study

550 women were excluded as they did not provide a response in confounding factors

**Supplementary Figure 1. Sample selection process**
